# Alzheimer’s research—breakthrough or breakdown?

**DOI:** 10.1093/braincomms/fcab217

**Published:** 2021-10-01

**Authors:** Tara L Spires-Jones

**Affiliations:** Centre for Discovery Brain Sciences; The University of Edinburgh, Edinburgh, UK

As someone who has been working in the field of Alzheimer’s disease research for the past 15 years, 2021 has been a doozy. Until this year, there were no approved disease-modifying drugs and almost two decades of very expensive clinical trial failures of amyloid lowering drugs despite many pre-clinical successes. On 7 June 2021, the Food and Drug Administration in the USA approved the anti-amyloid antibody aducanumab (marketed as Aduhelm) for the treatment of Alzheimer’s disease. On the one hand, having an approved drug that robustly clears amyloid pathology from the brain based on decades of strong fundamental science is something we should celebrate. On the other hand, the Food and Drug Administration’s accelerated approval decision went against a unanimous ‘no’ vote from its Advisory Committee and has sparked controversy in the field.

Aducanumab did not complete its two Phase 3 trials which were halted when an independent advisory committee found that the drug was not going to be effective in slowing cognitive decline. However, months later, Biogen announced that a change midway through one of the halted trials meant that a subset of participants had high exposure to the drug and that these people showed improvements. Most scientists who commented on the available data agreed that the drug lowers amyloid pathology, but there was much more scepticism surrounding the claim that cognitive decline was ameliorated. The Food and Drug Administration ruled despite this controversy that since aducanumab clears amyloid pathology, they will give Biogen 9 years to show that it also helps with cognition. The problem with this logic stems from the clear dissociation between amyloid in the brain and cognitive decline, and many have criticized the Food and Drug Administration for basing the decision on this surrogate biomarker without proper validation of its link to disease progression or symptoms.^1^ So now we have one provisionally approved disease-modifying treatment but there are so many problems with it that I do not know whether to celebrate or cry. And, we still need life-changing treatments for Alzheimer’s disease.

I have been thinking a lot about this lately and get asked by many, many people why it is taking so long to develop effective treatments. I think the biggest part of the answer is that the brain is phenomenally complex. We do not fully understand healthy brain function—how do the billions of neurons connected by trillions of synapses work together to make us human? It is thus no surprise that understanding how brain function is damaged by complex disease and how to stop this damage is a daunting and difficult task. Beyond this biological problem, there are some behavioural hurdles that have slowed progress in our field. Scientists are incentivized towards novel, positive results. We need ‘high-impact’ papers and completely new, shiny ideas for our funding and promotions. We are not usually rewarded for careful, incremental science that builds on the shoulders of giants.

One of the reasons I accepted the job of editor in chief of *Brain Communications* was to foster robust translational neuroscience studies that will hopefully lead to both broadening of our knowledge about how the brain functions and effective treatments for neurologic and psychiatric disorders. We do not require papers to be novel findings and welcome replication studies and well-substantiated negative results as these are important to advancing the field.

In this issue of *Brain Communications*, we have many examples of solid research in dementias, including very interesting tools and ideas ranging from characterizing new tau PET tracers and early signs predicting memory decline to using machine learning to tease out inter-individual differences underlying variability in disease symptoms.^2–4^ These studies and others in the field make me hopeful that even if the recent approval of a new drug is not the breakthrough we all hope it will be, we still have hugely exciting and important fundamental research coming through the pipeline that will move us towards the goal.

## Competing interests

The authors report no competing interests.
